# Meta-analysis of mucosal microbiota reveals universal microbial signatures and dysbiosis in gastric carcinogenesis

**DOI:** 10.1038/s41388-022-02377-9

**Published:** 2022-06-09

**Authors:** Changan Liu, Siu-Kin Ng, Yanqiang Ding, Yufeng Lin, Weixin Liu, Sunny Hei Wong, Joseph Jao-Yiu Sung, Jun Yu

**Affiliations:** 1 Institute of Digestive Disease and Department of Medicine and Therapeutics, State Key Laboratory of Digestive Disease, Li Ka Shing Institute of Health Sciences, CUHK Shenzhen Research Institute, The Chinese University of Hong Kong, Hong Kong, SAR China; 2 Lee Kong Chian School of Medicine, Nanyang Technological University, Singapore, Singapore

**Keywords:** Gastric cancer, Microbiology

## Abstract

The consistency of the associations between gastric mucosal microbiome and gastric cancer across studies remained unexamined. We aimed to identify universal microbial signatures in gastric carcinogenesis through a meta-analysis of gastric microbiome from multiple studies. Compositional and ecological profiles of gastric microbes across stages of gastric carcinogenesis were significantly altered. Meta-analysis revealed that opportunistic pathobionts *Fusobacterium*, *Parvimonas*, *Veillonella*, *Prevotella* and *Peptostreptococcus* were enriched in GC, while commensals *Bifidobacterium*, *Bacillus* and *Blautia* were depleted in comparison to SG. The co-occurring correlation strengths of GC-enriched bacteria were increased along disease progression while those of GC-depleted bacteria were decreased. Eight bacterial taxa, including *Veillonella*, *Dialister*, *Granulicatella*, *Herbaspirillum*, *Comamonas*, *Chryseobacterium*, *Shewanella* and *Helicobacter*, were newly identified by this study as universal biomarkers for robustly discriminating GC from SG, with an area under the curve (AUC) of 0.85. Moreover, *H. pylori*-positive samples exhibited reduced microbial diversity, altered microbiota community and weaker interactions among gastric microbes. Our meta-analysis demonstrated comprehensive and generalizable gastric mucosa microbial features associated with histological stages of gastric carcinogenesis, including GC associated bacteria, diagnostic biomarkers, bacterial network alteration and *H. pylori* influence.

## Introduction

Gastric cancer (GC) is the fifth most commonly diagnosed cancer, the fourth leading cause of cancer death, and responsible for 7.7% of all cancer-related deaths worldwide in 2020 [1]. It develops through a series of stages including superficial gastritis (SG), atrophic gastritis (AG), intestinal metaplasia (IM), dysplasia and gastric carcinoma [2]. It has been well known that *H. pylori* infection plays a primary role in gastric cancer development [3]. Infection with *H. pylori* is very prevalent, which has been estimated that at least 50% of adults harbour such infection worldwide [4]. However, only 1% to 3% develop gastric adenocarcinoma among infected individuals [5]. Moreover, successful eradication of *H. pylori* does not guarantee the prevention of gastric cancer development [6, 7]. Additionally, it is generally believed that *H. pylori* prefers a healthy stomach environment and that *H. pylori* colonization decreases at the later stages of carcinogenesis [8, 9]. These observations suggest that, besides *H. pylori*, other factors also contribute to gastric tumorigenesis [10].

*H. pylori* infection causes a decreased secretion of stomach acid, leading to the overgrowth of non-*H. pylori* microbes in the gastric ecological niche. Furthermore, *H. pylori* can cause the formation of bacterial biofilms, making it easier for oral bacteria to colonize in the stomach [11]. The association of *H. pylori* and other microbes in GC development was reported that gastric microbiota after *H. pylori* eradication could restore to a similar status of negative subjects [12]. Solid evidences have revealed the association between gastric commensal microbes other than *H. pylori* and the development of GC. Mice harboring a complex microbiota with *H. pylori* infection developed gastric cancer much faster than germ-free mice monocolonized with *H. pylori* [11]. Insulin–gastrin (INS-GAS) mice with a combination of three bacteria species or microbes complex could both result in gastritis, atrophy and dysplasia independent of *H. pylori* infection [11]. Thus, non-*H. pylori* commensals may contribute GC development, together with or independent of *H. pylori* infection. The potential interactions between *H. pylori* and gastric microbial communities, which may contribute to gastric carcinogenesis need to be further elucidated.

Several independent studies characterised the human gastric microbiota in gastric mucosa tissues from patients with GC and precancerous lesions using next-generation sequencing (NGS) of the bacteria 16 S rRNA gene [13–18]. However, the reproducibility and predictive accuracy of these microbial signatures identified independently in each study remain unclear. There is thus a need to perform a comprehensive and multi-cohort analysis to provide an unbiased and well-powered assessment of the link between gastric microbiota and gastric carcinogenesis. In this study, we carried out a meta-analysis of gastric microbiome in progressive stages of gastric tumorigenesis. We integrated and re-analysed raw 16 S rRNA gene sequence data from six independent studies across 825 gastric tissue biopsies. The microbial compositions and taxonomic alterations across stages of GC development were also examined. The robustness of the associations between microbiome and disease progression was assessed through multi-cohort comparisons. The bacterial biomarkers for classifications of different disease groups were identified and validated. We investigated the interactions between bacteria and taxonomic functions for each disease stage. In addition, we explored the effect of *H. pylori* on microbial communities.

## Results

### Mucosal microbiota differs across disease stages

**Fig. 1:**
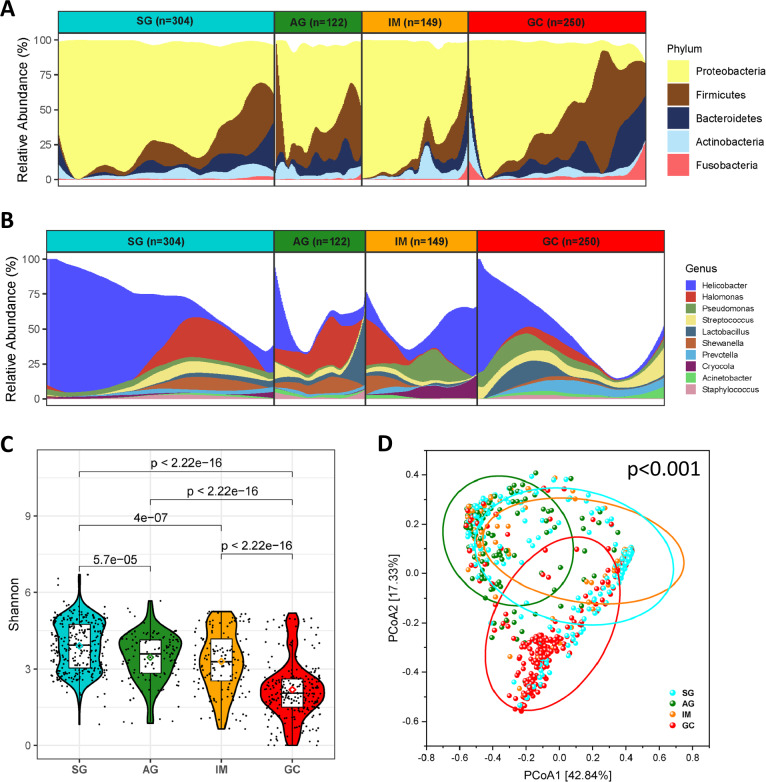
Microbiome data profiles across stages of gastric carcinogenesis. **A** Compositional bar plot for the relative abundance of top bacterial phyla across subjects in each stage. All the illustrated top 5 phyla with mean relative abundance >1%. **B** Compositional bar plot for the relative abundance of top bacterial genera across subjects in each stage. All the illustrated top 10 genera with mean relative abundance >1%. **C** Bacterial diversity (alpha diversity) estimated by Shannon index for patients in each group. The diamond symbols indicated the corresponding mean value for each group. Pairwise comparisons were performed using Wilcoxon rank-sum test. **D** Principal coordinate analysis (PCoA, beta diversity) for all the subjects. It was based on Bray-Curtis distance. *p*-value was estimated by permutational multivariate analysis of variance (PERMANOVA).

To explore the global microbial signature associated with gastric carcinogenesis, we collected and re-analysed 16 s rRNA sequencing data of 825 gastric biopsy samples from six independent studies. The dataset comprised patients from five ethnic groups in three continents, covering all four stages of gastric cancer (predominantly SG and GC). We first explored the overall microbial compositions along the progression of gastric cancer. Five phyla, *Proteobacteria*, *Firmicutes*, *Bacteroidetes*, *Actinobacteria* and *Fusobacteria*, dominated the gastric microbiota in descending order of overall relative abundance (Fig. 1A). The relative abundances of these phyla were significantly altered among the four disease stages (*p* < 0.05; Fig. S1A). At genus level, the gastric mucosal microbiota was dominated by 10 genera, including *Helicobacter*, *Halomonas*, *Pseudomonas*, *Streptococcus*, *Lactobacillus*, *Shewanella*, *Prevotella*, *Acinetobacter*, *Cryocola*, and *Staphylococcus* (Fig. 1B). Similar to the dominant phyla, the relative abundances of these 10 dominant genera were also significantly different among disease progression (*p* < 0.0001; Fig. S1B). The abundance of *Helicobacter* was significantly higher in SG than in other disease stages (*p* < 2e−16; Fig. S1B). To assess the alterations in the microbial communities among different disease stages, we measured the alpha diversity (within samples) and beta diversity (between samples). Through evaluating alpha diversity using the Shannon index, we found that the gastric cancer group had the lowest microbial diversity compared with SG, AG and IM (*p* < 2.22e−16; Fig. 1C). Consistent results were observed using Chao1 (Fig. S2A) and Simpson indices (Fig. S2B). In addition, the microbial diversities exhibited a descending trend along the disease progression (Fig. 1C). Beta diversity was visualized by principal coordinate analysis (PCoA) based on Bray-Curtis distance. The diversity captured by the top two principal coordinates was around 60%. The microbial compositions of the four disease stages were significantly different (*p* < 0.001; Fig. 1D). Consistent findings were obtained using unweighted UniFrac (Fig. S2C) and weighted UniFrac diatances (Fig. S2D).

### Bacterial biomarkers for distinguishing GC from SG

**Fig. 2:**
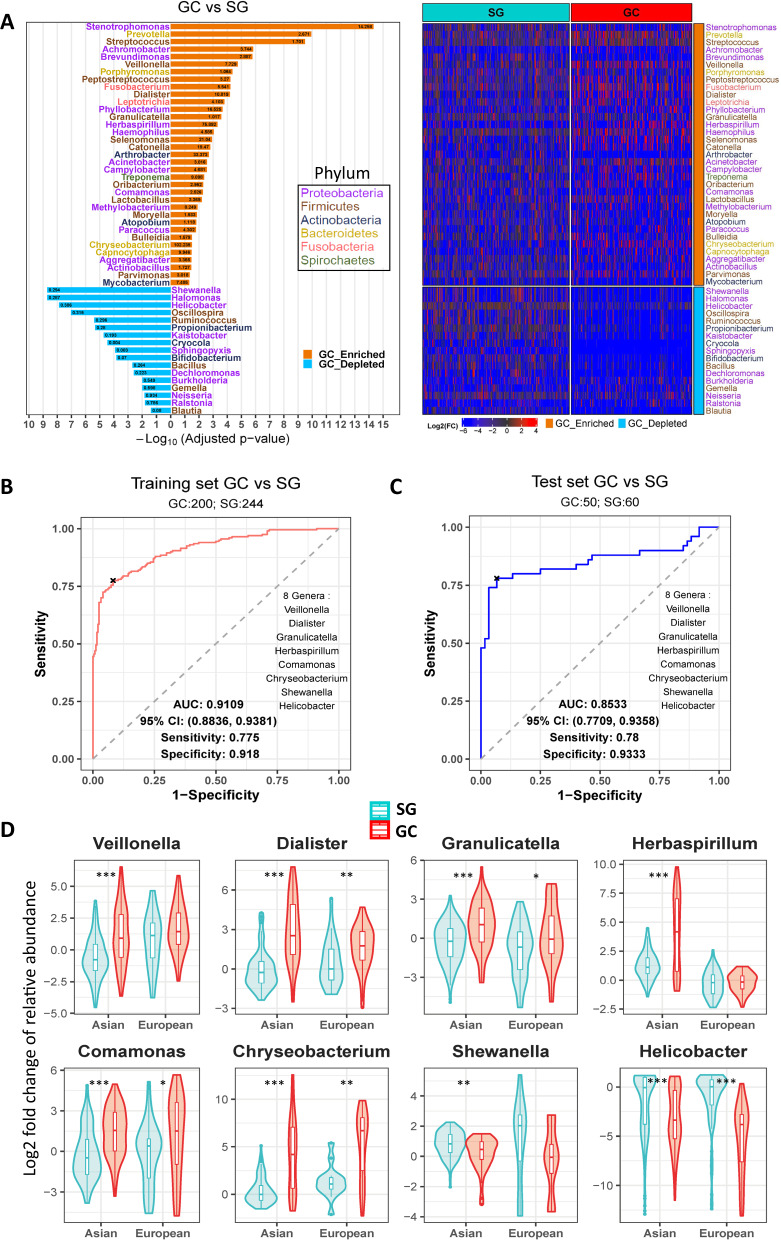
Differentially abundant bacteria between GC and SG and the diagnostic genera markers. **A** Mirror bar plot (left panel) and heatmap (right panel) for the significant differentially abundant genera between GC and SG. Numbers in the bar plot were the corresponding fold changes of means of relative abundances for GC vs SG. The significantly altered genera were determined by MaAsLin2 with adjusting age, gender and *H. pylori* status. Adjusted *p*-value <0.05 as the cut-off for significance. **B** Receiver operating characteristic (ROC) analysis for the 8 genera markers with logistic regression model discriminating GC from SG on training set. **C** Receiver operating characteristic analysis for the same logistic regression model discriminating GC from SG in test set. The 8 genera markers were determined by backward stepwise selection algorithm from the significantly altered genera. The ratio of sample size of training set to that of test set was 8:2. **D** Violin graph for the Log2 fold change of relative abundance of the 8 genera markers between GC and SG in different ethnic groups. Significance was obtained by Wilcoxon rank-sum test (*p < 0.05, **p < 0.01, ***p < 0.001).

To determine the significantly altered genera between GC and SG, we built general linear models with study, age, gender and *H*. *pylori* status adjusted using MaAsLin2. Among 52 bacterial genera significantly different between the two stages, 35 genera were enriched in GC compared with SG including *Veillonella, Fusobacterium, Prevotella, Stenotrophomonas*, *Streptococcus* and *Lactobacillus*, whereas 17 were depleted including *Shewanella*, *Halomonas*, *Helicobacter*, *Bifidobacterium*, *Bacillus* and *Blautia* (Fig. 2A). Most of the identified bacterial genera belonged to the phylum *Proteobacteria*. Since the sample sizes in the original studies were unequal, we further investigated the impact of samples sizes on the differential abundant analysis. By analyzing resampled datasets with a matched sample size, the abundance of these 52 genera were found significantly altered between GC and SG in almost all resampled datasets, suggesting the robustness of differential abundant analysis (Fig. S3). We then assessed the 52 significantly altered bacterial genera for their potential as diagnostic biomarkers for discriminating GC from SC. Six GC-enriched (*Veillonella*, *Dialister*, *Granulicatella*, *Herbaspirillum*, *Comamonas*, *Chryseobacterium*) and two GC-depleted genera (*Shewanella* and *Helicobacter*) were identified as potential biomarkers using the backward stepwise selection algorithm. A logistic regression model was built based on the eight biomarkers. To evaluate the performance of the model, receiver operating characteristic (ROC) analysis was conducted, yielding an area under the curve (AUC) of 0.9109 for the training set (Fig. 2B) and 0.8533 for the test set (Fig. 2C) respectively. Additionally, we explored the differences of relative abundances between GC and SG for the eight diagnostic biomarkers in two different populations (Asian and European). Except for *Veillonella*, *Herbaspirillum* and *Shewanella*, all the evaluated biomarkers were significantly altered between GC and SG in both populations (Fig. 2D).

### Bacterial biomarkers to distinguish other lesions

We further studied the differentially abundant bacterial genera and diagnostic biomarkers for AG vs SG, IM vs SG, IM vs AG and GC vs IM. The bacterial taxa significantly altered between AG and SG included 28 AG-enriched genera and 7 AG-depleted genera (Fig. S4A). Among these 35 taxa, nine genera were capable of discriminating samples between AG and SG by achieving an AUC of 0.8763 and 0.8611 on training (Fig. S4B) and test sets (Fig. S4C) respectively. Similarly, 12 genera (5 enriched and 7 depleted in IM) were found to be significantly altered between IM and SG. 5 genera were enriched in IM, while 7 genera were IM-depleted (Fig. S4D). We selected 10 genera to construct a classification model that was capable of discriminating IM from SG, with an AUC of 0.7117 and 0.7075 in training (Fig. S4E) and test sets (Fig. S4F) respectively. For AG vs SG, IM vs SG and GC vs SG, all the diagnostic biomarkers were cross-validated with support vector machine (SVM) model using the same training and test sets. The performance of these two classification models was similar (Fig. S5). For IM vs SG, 4 IM-enriched genera and 31 IM-depleted genera were significantly altered (Fig. S6A). Four genera were capable of discriminating samples between IM and SG by achieving an AUC of 0.8703 and 0.842 on training (Fig. S6B) and test sets (Fig. S6C) respectively. The bacterial taxa were significantly altered between GC and IM included 23 GC-enriched genera and 14 GC-depleted genera (Fig. S6D). Four genera were capable of discriminating samples between GC and IM by achieving an AUC of 0.8864 and 0.8766 on training (Fig. S6E) and test sets (Fig. S6F) respectively. Similarly, the diagnostic biomarkers for IM vs AG and GC vs IM were cross-validated with support vector machine (SVM) models using the same training and test sets, yielding the consistent performances (Fig. S7). Moreover, 79 bacteria were collected by combining the significantly altered genera for GC vs SG, AG vs SG, IM vs SG, IM vs AG and GC vs IM. The heatmap for the 79 bacteria with differential abundance at each disease stage was illustrated in Fig. S8.

### Alteration of bacteria correlations along stages of GC progression

**Fig. 3:**
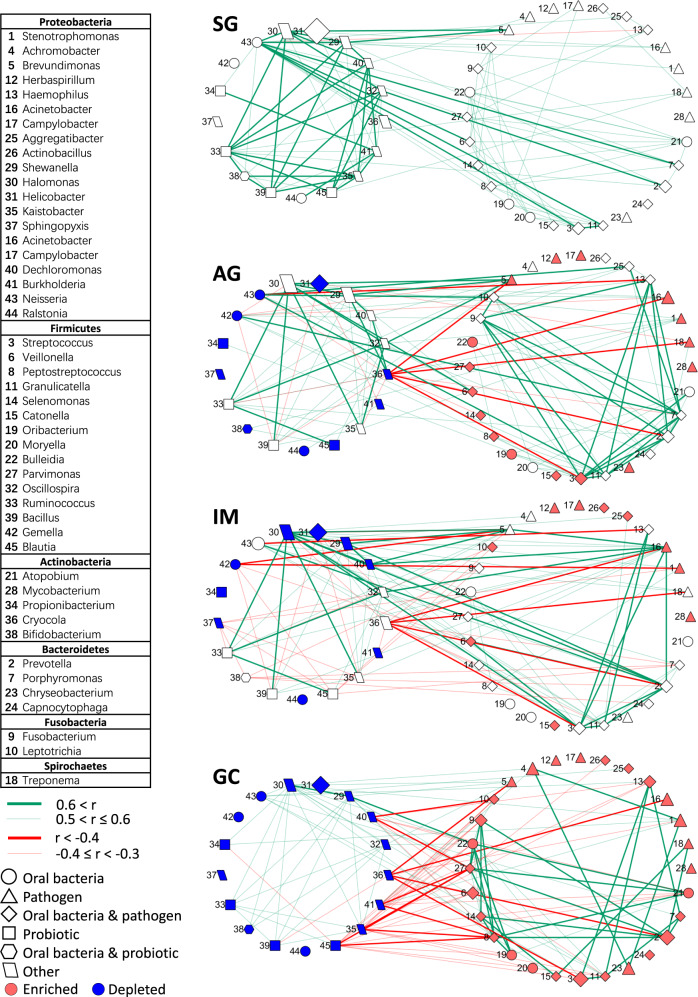
Correlation networks of gastric cancer associated bacteria with disease progression. Correlation strengths were estimated by SparCC algorithm. Significant correlations with adjusted *p*-value < 0.05 were remained for visualization. Bacteria in the left circle were GC-depleted compared with SG while those in the right circle were GC-enriched. The enriched bacteria compared with SG were labelled with red colour while the depleted ones were labelled with blue colour in each disease stage. The sizes of nodes were proportional to the median of relative abundance of corresponding genera in each stage respectively.

To explore the interaction among diseases-associated bacteria (GC-enriched and depleted genera) along the progression of gastric cancer, we estimated their correlations using SparCC algorithm. We found that the distributions of correlations were significantly altered between GC and all three benign disease stages (*p* < 0.001, Fig. S9). Moreover, the positive correlations among GC-enriched bacteria strengthened progressively along disease progression, especially the correlations of *Fusobacterium* with *Prevotella*, *Parvimonas*, *Peptostreptococcus* and *Streptococcus* (Fig. 3). By contrast, the co-occurring correlation (positive ones) strengths of GC-depleted bacteria were steadily decreased along GC development, including the correlations of *Bifidobacterium* with *Bacillus* and *Ruminococcus*. Additionally, co-excluding correlations (negative ones) among GC-enriched and GC-depleted bacteria were strengthened with disease progression, such as the interactions between *Blautia* and *Parvimonas* or *Peptostreptococcus*.

### Shift in microbial function across stages of gastric carcinogenesis

**Fig. 4:**
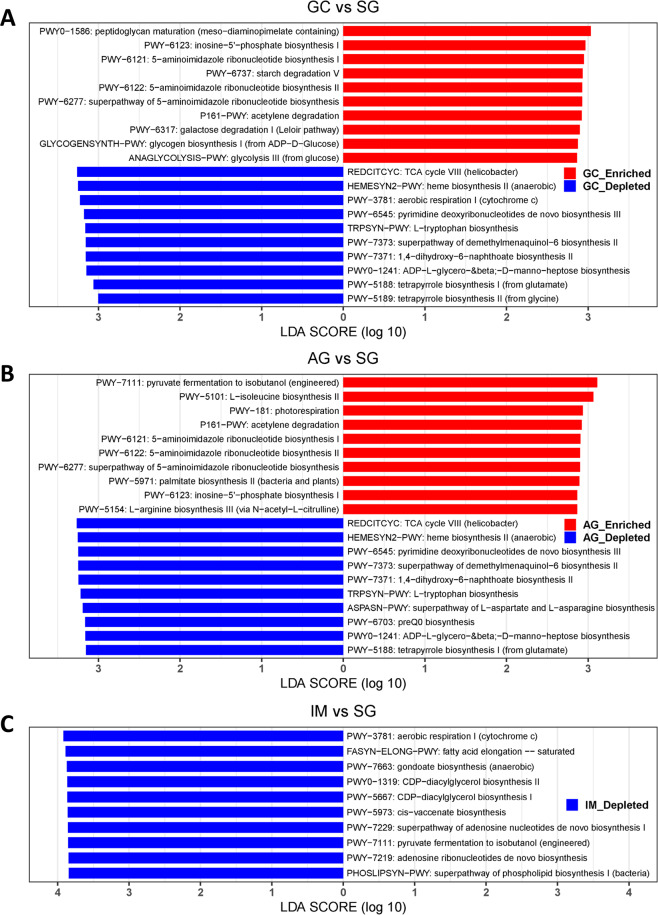
Predicted microbiota functional changes between stages of gastric cancer in MetaCyc pathways. **A** Bar plot of the top 10 significantly altered MetaCyc pathways between GC and SG. **B** Bar plot of the top 10 significantly altered MetaCyc pathways between AG and SG. **C** Bar plot of the top 10 significantly altered MetaCyc pathways between IM and SG. Significance was determined by Linear discriminant analysis (LDA) effect size (LEfSe) method with cutoff LDA score >2 and *p*-value < 0.05.

We applied PICRUSt2 to infer the functional potential of the gastric mucosal microbiome. Compared with SG, the most significant MetaCyc pathway enriched in GC was peptidoglycan maturation (meso-diaminopimelate containing) of peptidoglycan biosynthesis. Moreover, the pathways related to purine nucleotide biosynthesis, such as inosine-5’-phosphate biosynthesis and 5-aminoimidazole ribonucleotide biosynthesis, were also enriched in GC. Other GC-enriched pathways are related to carbohydrate degradation and biosynthesis, including starch degradation V, galactose degradation I (Leloir pathway), and glycogen biosynthesis I (from ADP-D-Glucose) (Fig. 4A). Interestingly, MetaCyc pathway involved in *Helicobacter* specific tricarboxylic acid cycle (TCA cycle VIII) was the most depleted pathway in GC (Fig. 4A and Table S1) as well as in AG (Fig. 4B and Table S2) in comparison to SG. The pathway TCA cycle VIII (*helicobacter*) was also significantly depleted in IM compared with SG (Fig. 4C and Table S3). Additionally, we correlated the top altered MetaCyc pathways with significantly altered microbes for GC vs SG in each disease stage (Fig. S10). We further investigated the significantly altered KEGG pathways between disease stages. We found that the epithelial cell signaling pathway in *H. pylori* infection (ko05120) was significantly depleted in AG, IM and GC compared with SG (Fig. S11, Tables S4–S6). All the significantly altered pathways were provided in Tables S1–S6.

### The influence of *H. pylori* on the microbial community

**Fig. 5:**
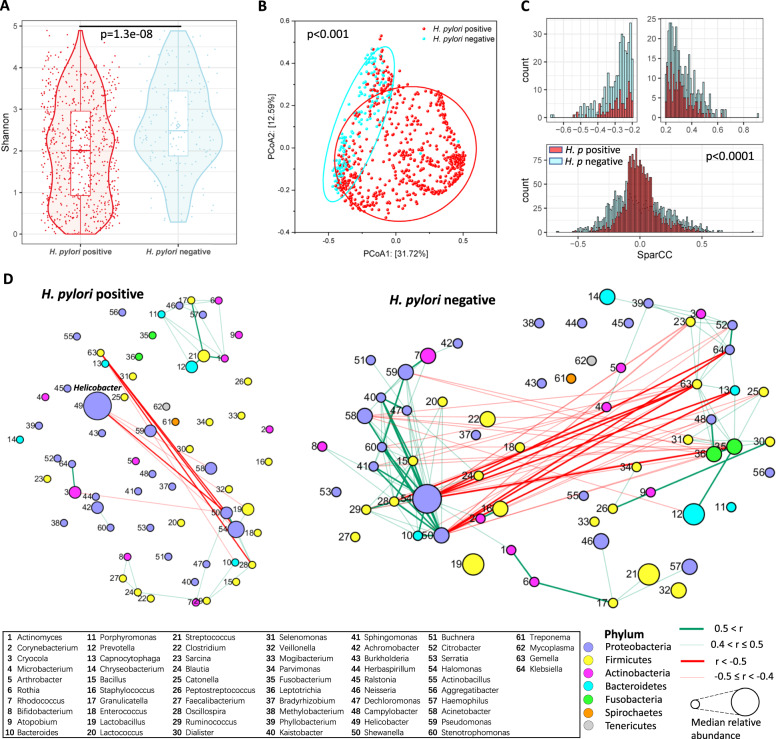
The influence of *H. pylori* in the microbiota community. **A** Bacterial diversity (Alpha diversity) estimated by Shannon index for patients with different *H. pylori* status. *p*-value was obtained by Wilcoxon rank-sum test. **B** Principal coordinate analysis (PCoA, Beta diversity) for subjects in different *H. pylori* status. It was based on Bray-Curtis distance. *p*-value was estimated by permutational multivariate analysis of variance (PERMANOVA). **C** Histograms of the distributions of SparCC correlation strengths for abundant bacteria with different *H. pylori* status. Genera with median of relative abundance >0.1% were considered as abundant bacteria. *p*-value was obtained by Kolmogorov-Smirnov test. **D** Correlation networks of abundant bacteria with different *H. pylori* status. Correlation strengths were estimated by SparCC algorithm. Significant correlations with adjusted *p*-value < 0.05 were remained for visualization. The sizes of nodes were proportional to the median of relative abundance of corresponding genera in each *H. pylori* status.

We further investigated the effect of *H. pylori* status on gastric cancer microbiome structure. Overall, the alpha diversity evaluated based on Shannon index revealed that the gastric microbiome in the *H. pylori*-negative patients had significantly higher microbial diversity (*p* = 1.3e−08; Fig. 5A). Moreover, the microbial compositions between *H. pylori*-negative and *H. pylori*-positive groups were significantly different as reflected by beta diversity based on Bray-Curtis distance (*p* < 0.001; Fig. 5B). We then explored the influence of *H. pylori* on microbial interactions using SparCC algorithm. The distribution of bacteria-bacteria correlations between these two groups were significantly different (*p* < 0.0001; Fig. 5C). Higher numbers of strong co-excluding and co-occurring interactions (|r| > 0.5, adjusted *p*-value < 0.05) among bacteria were observed in *H. pylori*-negative group compared with *H. pylori*-positive group (Fig. 5D).

We finally explored the effect of *H. pylori* in each disease stage. The *H. pylori*-negative group exhibited a higher microbial diversity in SG and IM. A similar trend was observed in AG and GC, though it did not reach a significant level (Fig. S12A). The beta diversities between the two groups were significantly altered in all four disease stages (Fig. S12B). For the bacteria interactions - within each disease group, significantly alterations of correlation distributions were observed. Consistent with the global state, the number of strong associations was higher in *H. pylori*-negative group (Fig. S12C).

## Discussion

Gastric cancer is a multifactorial disease involving the interactions among host, microbial and environmental factors, despite *H. pylori* being recognized as the crucial risk factor. Microbiome dysbiosis has been shown to associate with many gastrointestinal diseases including cancers [19]. Our meta-analysis demonstrated that gastric microbiota was dominated by the phyla *Proteobacteria*, *Firmicutes*, *Bacteroidetes*, *Actinobacteria* and *Fusobacteria*, which is consistent with previous findings [16, 17, 20]. The microbial diversity and richness were significantly decreased in carcinoma compared with precancerous stages, in accordance with some previous studies [16, 18]. There are several conflicting reports about the changes in the alpha diversity of the gastric microbiome across GC cascade [13, 14, 21]. The inconsistency may be due to limited sample size and the indices applied to evaluate the diversity. Reduced microbial diversity has been recognized as a characteristic of disease status, including inflammatory diseases and cancers [19–21].

At the genus level, we identified some significant alterations of bacteria abundance across disease stages. We found that more than half of the GC-enriched bacteria were commonly identified in the oral cavity compared with SG, including *Prevotella*, *Streptococcus*, *Fusobacterium*, *Veillonella*, *Peptostreptococcus* and *Parvimonas*. The enrichment of oral bacteria have been reported in several diseases, such as inflammatory bowel diseases, colorectal cancer and pancreatic cancer [21–26]. Periodontal diseases caused by oral microbiota dysbiosis were linked to gastric carcinoma as suggested by some studies [27, 28]. *Fusobacterium* species have drawn a lot of attention due to their pro-inflammatory nature [29, 30]. Particularly, a species of *Fusobacterium* has been shown to potentiate intestinal tumorigenesis and modulate the tumor-immune microenvironment, indicating a potential as a diagnostic biomarkers for colorectal cancer [31, 32]. Some studies revealed that *Streptococcus* species were associated with oesophageal cancer through inducing inflammatory cytokines in oesophageal epithelial cells [33, 34]. *Veillonella* species were found to be increased in oral, lung and colorectal cancer patients [32–37], suggesting its potential role in tumorigenesis of GC. The overabundance of *Prevotella* species at mucosal sites was suggested to be associated with some localized and systemic diseases, including periodontitis, bacterial vaginosis, rheumatoid arthritis and low-grade systemic inflammation [38]. *Peptostreptococcus stomatis* and *Parvimonas micra* in feces have been found related to colorectal cancer [32]. Multiple evidences suggest that the GC-depleted taxa *Bifidobacterium*, *Bacillus* and *Blautia* might be putative probiotics. *Bifidobacterium longum* was reported to exhibit anti-proliferation and anti-angiogenesis effect against gastric cancer by downregulating COX2 expression [39]. Moreover, clinical trials were conducted using *Bifidobacterium* as probiotic supplement with antibiotics and proton pump inhibitors to eradicate *H. pylori* [40]. The antagonistic activity of *Bacillus* spp. has been explored against large number of pathogens. One study also reported the anti *H. pylori* activity of tested probiotic *Bacillus subtilis* strains, which was attributed to the secretion of aninocoumacin A antibiotic [41]. Anti-inflammatory effects was also demonstrated for *Blautia* in gastrointestinal diseases, including inflammatory bowel diseases and intestinal graft-versus-host disease [42]. The putative pathogenic or probiotic functions of the bacterial taxa identified in the meta-analysis were supported by several studies [13, 14, 16, 18], therefore their functional roles in GC tumorigenesis merit further investigation.

The most relevant genera that characterised each disease stage identified by our meta-analysis allowed us to discover robust diagnostic biomarkers, which showed excellent performance. The 8 bacterial biomarkers were shown to be efficient (AUC of 0.85) in distinguishing GC from SG on a multi-cohort dataset, underlying their implications in disease progressive as well as their clinical applications. Besides, we found 9 and 10 bacterial biomarkers capable of classifying AG from SG (AUC of 0.86) and IM from SG (AUC of 0.71). The comparable AUC values obtained by different classification models (logistic regression vs SVM) indicated that the bacterial biomarkers were robust regardless of models used.

Changes in bacterial correlations could partially explain gastric tumorigenesis and reflect disease-specific microenvironment. We found that the sub-network formed by GC-enriched bacteria included many opportunistic pathogens, including *Stenotrophomonas*, *Streptococcus*, *Fusobacterium*, *Parvimonas Peptostreptococcus* and *Prevotella*. By contrast, for the sub-network generated by GC-depleted genera, there were some putative probiotics, such as *Bifidobacterium*, *Bacillus*, and *Blautia*. We observed increasing strengths of co-occurring correlations among GC-enriched bacteria implied that they could be more active in the later disease stage and therefore contribute to gastric carcinogenesis. We also observed decreasing strengths of co-occurring interactions among GC-depleted bacteria along disease progression, suggesting that they potentially play an essential role in maintaining the balanced composition of gastric microbiota. Moreover, the increase of negative correlation strengths between GC-enriched and GC-depleted bacteria suggested the possibility of reciprocally antagonistic effects between them. The alteration of network structure for the gastric microbial community with GC development was also reported by other researchers. One study found that the correlation strengths of GC-enriched and GC-depleted bacteria increased with disease progression [43]. Another study showed that strong co-excluding interactions in gastric microbiota between *Helicobacter* and *Fusobacterium*, *Neisseria*, *Prevotella*, *Veillonella*, *Rothia* were found only in patients with advanced gastric lesions, and were absent in normal/superficial gastritis group [43]. All the findings suggest that alteration in the gastric microbial community may have implications in gastric tumorigenesis.

We further addressed the functional features of gastric microbiota across disease stages. Compared with SG, we observed the most significant pathway enriched in GC was related to peptidoglycan biosynthesis. Peptidoglycan plays an important role in modulating host inflammatory response to *H. pylori* infection, allowing the bacterium to persist and induce carcinogenic consequences in the gastric niche [43]. Moreover, we found that some pathways enriched in GC involve in purine nucleotide biosynthesis. Studies showed that some enzymes involved in de novo purine biosynthesis promote GC development [44, 45], such as U2AF homology motif kinase 1 (UHMK1) and phosphoribosylaminoimidazole carboxylase, phosphoribosylaminoimidazole succinocarboxamide synthetase (PAICS). Interestingly, the second top GC-enriched pathway compared with SG was related to inosine-5′-monophosphate (IMP) biosynthesis, which also involves in purine nucleotide biosynthesis. Inosine-5′-monophosphate dehydrogenase (IMPDH) is a purine biosynthetic enzyme that catalyzes the nicotinamide adenine dinucleotide (NAD+)-dependent oxidation of inosine-5′-monophosphate to xanthosine monophosphate (XMP), the first rate-limiting step towards the de novo biosynthesis of guanine nucleotides from IMP. Guanine nucleotide synthesis is essential for maintaining normal cell function and growth. IMPDH expression is found to be upregulated in some tumour tissues and cancer cell lines. Therefore, IMPDH has been addressed as a drug target for cancer chemotherapy [46].

Furthermore, we explored the effects of *H. pylori* on the diversity and interactions of microbial communities for all involved subjects and each disease stage. We observed a decrease in richness of microbiome in *H. pylori*-positive patients, which is consistent with previous reports [18, 47]. This observation was still held for disease stage SG and IM. As for beta diversity, the significant difference was also demonstrated between these two *H. pylori* status. Additionally, we observed the significant decrease of interactions among bacteria in *H. pylori*-positive group in all participants and each disease group. These findings were supported by the previous study [18]. The dysbiosis may be caused by *H. pylori* infection and colonization. During colonization, the adhesins produced and virulence factors delivered by *H. pylori* may weaken the interactions among other bacteria. All these indicate *H. pylori* with the capacity to alter gastric microbiota community dramatically.

In conclusion, we assessed the gastric microbiome using multi-cohort datasets and identified biomarkers capable of distinguishing patients across disease stages. The increase in the abundance of opportunistic pathogens (*e.g. Veillonella* and *Parvimonas*) concomitant with the decrease in putative probiotics (e.g. *Bifidobacterium*) was observed along the stages of disease progressive, as revealed by corresponding ecological and functional shifts in bacterial community. We found that the co-occurring correlation strengths of GC-enriched bacteria were increased while those of GC-depleted bacteria were decreased with GC progression. In the meanwhile, co-excluding correlations among GC-enriched and GC-depleted bacteria were strengthened. The top GC-enriched pathways were related to peptidoglycan biosynthesis and purine nucleotide biosynthesis. Additionally, we showed that *H. pylori* could modulate gastric microbiota, leading to reduction in microbial diversity and interactions among gastric microbes. Our meta-analysis provides additional insight into the functional involvements and therapeutic targets of the gastric bacteria other than *H. pylori* in contributing to gastric tumorigenesis.

## Materials and methods

### Study sample inclusion and data acquisition

In this meta-analysis, raw 16 S rRNA gene sequence data of 825 gastric tissue biopsies were integrated from six studies. The demographic and clinical details of included subjects were showed in Table 1. For the study Coker_2018 [18], 311 gastric biopsy samples of Chinese patients were included after removing 37 adjacent non-cancerous samples. Among the 404 gastric biopsy samples of Chinese subjects obtained from the study Sung_2019 [15], only 202 pre-treatment samples were used in our analysis. For the study Ferreira_2018 [16], raw data of 135 gastric biopsy samples from Portuguese patients was retrieved form Sequence Read Archive (SRA) under accession PRJNA413125. For the study Yu_2017 [17], raw data of 80 gastric tumor samples from China and 54 from Mexico was downloaded from SRA with identifier PRJNA310127. We included all the 31 gastric biopsy samples collected in South Korea from the study Eun_2014 [14]. The raw sequence data was available in the SRA under accession PRJNA239281. We fetched the raw data of gastric biopsy samples from 12 Malaysian patients with gastric cancer for the study Castano-Rodriguez_2017 [13] from European Nucleotide Archive (ENA) under accession PRJEB21497.

**Table 1:** Demographic and clinical details of subjects in each study.

Study	Characteristics	SG (*n* = 304)	AG (*n* = 122)	IM (*n* = 149)	GC (*n* = 250)	*p*-value
Coker_2018 (Chinese)	Sample size (*n* = 311)	110	117	45	39	
	Age	49.6 (10.9)	53.9 (14.0)	54.4 (10.3)	59.9 (10.7)	<0.001
	Gender					
	Male	56 (50.9%)	62 (53.0%)	15 (33.3%)	28 (71.8%)	0.006
	Female	54 (49.1%)	55 (47.0%)	30 (66.7%)	11 (28.2%)	
	*H. pylori* status					
	Positive	81 (73.6%)	82 (70.1%)	27 (60.0%)	27 (69.2%)	0.42
	Negative	29 (26.4%)	35 (29.9%)	18 (40.0%)	12 (30.8%)	
Sung_2019 (Chinese)	Sample size (*n* = 202)	103	5	94	–	
	Age	52.1 (8.3)	52.6 (7.5)	52.6 (8.2)	–	0.819
	Gender					
	Male	53 (51.5%)	1 (20.0%)	51 (54.3%)	–	0.359
	Female	50 (48.5%)	4 (80.0%)	43 (45.7%)	–	
	*H. pylori* status					
	Positive	103 (100%)	5 (100%)	94 (100%)	–	NA
	Negative	0 (0%)	0 (0%)	0 (0%)	–	
Ferreira_2018 (Portuguese)	Sample size (*n* = 135)	81	–	–	54	
	Age	43.6 (7.0)	–	–	58.8 (13.2)	<0.001
	Gender					
	Male	79 (97.5%)	–	–	32 (59.3%)	<0.001
	Female	2 (2.5%)	–	–	22 (40.7%)	
	*H. pylori* status					
	Positive	80 (98.8%)	–	–	47 (87.0%)	0.007
	Negative	1 (1.2%)	–	–	7 (13.0%)	
Yu_2017 (Chinese: 80 Mexican: 54)	Sample size (*n* = 134)				134	
	Age				61.8 (10.6)	NA
	Gender					
	Male	–	–	–	92 (68.7%)	NA
	Female	–	–	–	42 (31.3%)	
	*H. pylori* status					
	Positive	–	–	–	80 (59.7%)	NA
	Negative	–	–	–	54 (40.3%)	
Eun_2014 (Korean)	Sample size (*n* = 31)	10	–	10	11	
	Age	50.4 (11.5)	–	57.5 (7.3)	65.7 (11.3)	0.019
	Gender					
	Male	4 (40.0%)	–	7 (70.0%)	6 (54.5%)	0.445
	Female	6 (60.0%)	–	3 (30.0%)	5 (45.5%)	
	*H. pylori* status					
	Positive	7 (70.0%)	–	4 (40.0%)	7 (63.6%)	0.433
	Negative	3 (30.0%)	–	6 (60.0%)	4 (36.4%)	
Castano- Rodriguez_ 2017 (Malaysian)	Sample size (*n* = 12)	–	–	–	12	
	Age	–	–	–	62.1 (13.8)	NA
	Gender					
	Male	–	–	–	4 (33.3%)	NA
	Female	–	–	–	8 (66.7%)	
	*H. pylori* status					
	Positive	–	–	–	11 (91.7%)	NA
	Negative	–	–	–	1 (8.3%)	

Age format: mean (standard deviation). Kruskal-Wallis test was performed on continuous factor age for getting the *p*-values. Fisher’s exact test was performed on categorical factors gender and *H. pylori* status for obtaining the *p*-values.

*SG* superficial gastritis, *AG* atrophic gastritis, *IM* intestinal metaplasia, *GC* gastric cancer, *NA* not applicable.

### 16 S rRNA gene sequence data analysis

The 16 S rRNA gene sequence analysis was conducted using QIIME 2 (version 2020.11.0) [48]. Raw paired-end reads were joined by vsearch join-pairs. The joined sequences with more than 1 position (--p-quality-window=1) with quality score <15 (--p-min-quality=15) were discarded using quality-filter q-score command, and subsequently denoised using Deblur workflow to reduce sequencing errors and remove chimera reads. The resulting sequences were taxonomically assigned based on the Greengenes database (version 13.8) using BLAST + consensus taxonomy classifier with default settings. Microbial community analysis was conducted using vegan package in R. The richness and abundance of species in each sample (alpha diversity) were estimated by Shannon’s index. Dissimilarity of microbial communities among samples (beta diversity) was measured by Bray-Curtis distance and visualized with principal coordinate analysis (PCoA). Permutational multivariate analysis of variance (PERMANOVA) using Bray-Curtis distance with 1000 permutations was used to compare community dissimilarity of sample groups.

### Determination of differentially abundant bacteria

The differentially abundant bacteria at genus level among different sample groups were determined by MaAsLin2 (Microbiome Multivariable Associations with Linear Models, Maaslin2 R package) with study, age, gender and *H*. *pylori* status adjusted according to clinical details of included subjects. The significance criteria were adjusted *p*-value < 0.05, mean relative abundance >0.1% and prevalence >20%.

### Identification and validation of diagnostic biomarkers

For each disease stage (i.e. SG), data was randomly split into training and test sets in a ratio of 8:2. The optimal biomarkers for discriminating any two disease stages (i.e. GC vs SG) on training set were iteratively selected from differentially abundant bacteria based on the Akaike Information Criteria (AIC) using backward stepwise selection algorithm from MASS package in R. Logistic regression models were built using the selected bacterial biomarkers with the function “glm” from stats package, and their performance was evaluated on the corresponding test set. In addition to logistic regression model, we evaluated the selected bacteria biomarkers in discriminating disease stages using support vector machine (SVM) model on the same training set and test set as implemented in caret R package. The receiver operating characteristic (ROC) analysis was performed to illustrate the performances of classification models using pROC R package.

### Microbial correlation network analysis

SparCC algorithm was used to estimate the correlations between taxa from sparse compositional data. The empirical *p*-values of correlation coefficients were estimated based on 100 iterations. The correlation coefficients with adjusted *p*-values < 0.05 were considered significant and visualized with Cytoscape (version 3.7.2).

### Prediction of metagenomic functions

Functional prediction was performed using PICRUSt2 [49]. Predicted functional genes were categorised into MetaCyc metabolic pathways and Kyoto Encyclopedia of Genes and Genomes (KEGG) pathways. Significantly altered pathways between disease stages were determined by linear discriminant analysis (LDA) effect size (LEfSe) method [50] with a cutoff LDA score > 2 and *p*-value < 0.05.

### Statistical analyses

Pairwise comparison was performed using two-sided Wilcoxon rank-sum test (Mann-Whitney *U* test). Kruskal-Wallis test was used to compare multiple groups. Fisher’s exact test was performed on categorical variables (gender and *H. pylori* status). Kolmogorov-Smirnov test was applied to compare the distributions of correlation coefficients between bacteria for different sample groups. Benjamini-Hochberg false discovery rate correction was applied to adjust *p*-value for multiple tests. All the related statistical analyses were performed using R software (version 4.0.4).

## Supplementary information

Figure S1

Figure S2

Figure S3

Figure S4

Figure S5

Figure S6

Figure S7

Figure S8

Figure S9

Figure S10

Figure S11

Figure S12

Table S1

Table S2

Table S3

Table S4

Table S5

Table S6

## Data Availability

Raw sequence data for study Ferreira_2018 are available at Sequence Read Archive (SRA) under accession number PRJNA413125. Raw sequence data for study Yu_2017 are available at SRA under accession number PRJNA310127. Raw sequence data for study Eun_2014 are available at SRA under accession number PRJNA310127. Raw sequence data for study Castano-Rodriguez_2017 are available at European Nucleotide Archive (ENA) under accession number PRJEB21497. Raw sequence data for study Coker_2018 and study Sung_2019 are available from the corresponding author, Professor Jun Yu, upon reasonable request.
