# Supplementary material for: Meta-analysis of mucosal microbiota reveals universal microbial signatures and dysbiosis in gastric carcinogenesis
Source: Oncogene. 2022 Jun 9;41(28):3599–610. doi: 10.1038/s41388-022-02377-9 (PMC9270228; doi:10.1038/s41388-022-02377-9)
Supplement: Supplementary file 1 — Figure S1 [file 41388_2022_2377_MOESM1_ESM.pdf]

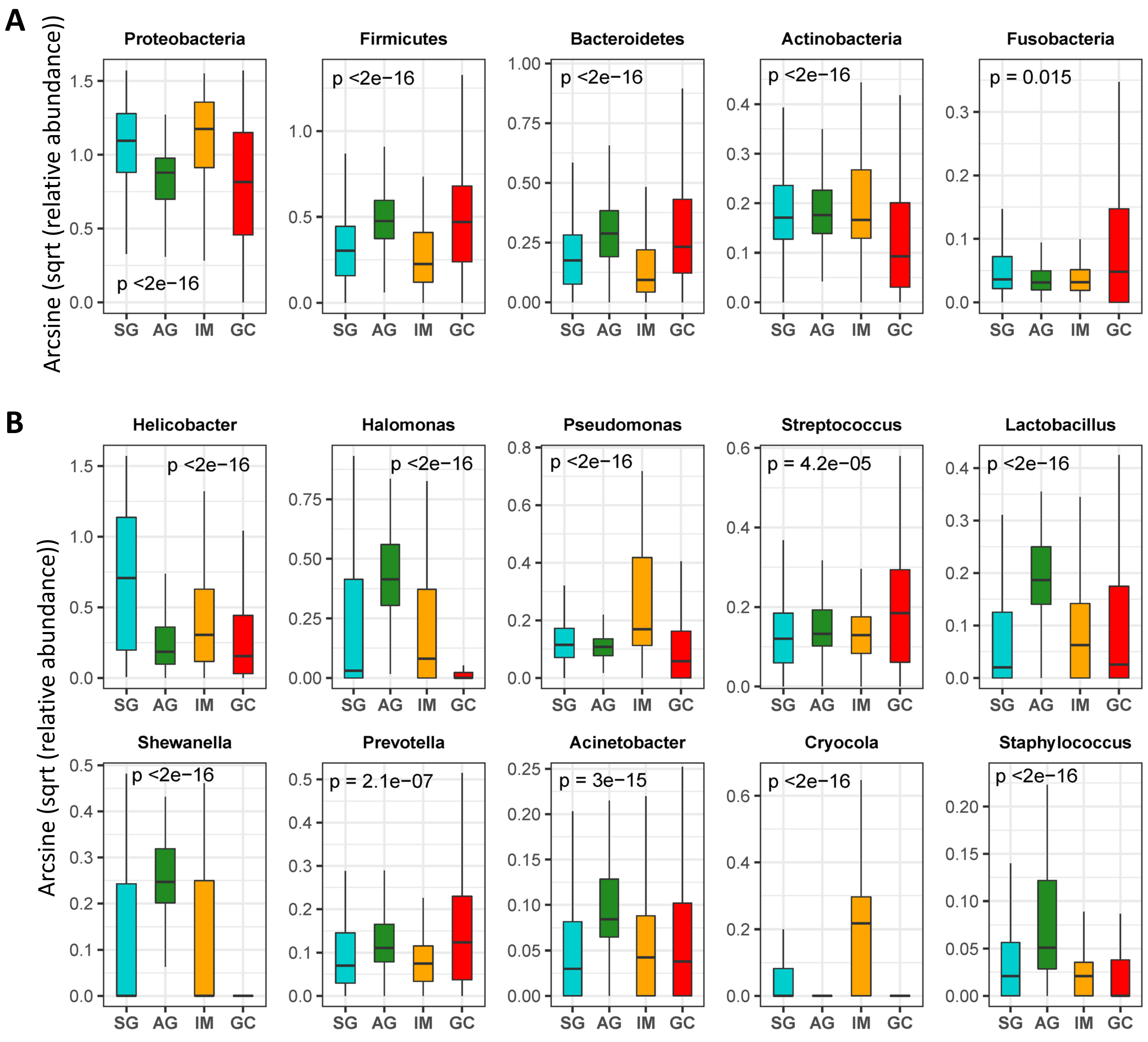

Figure S1. Box plots of the top abundant bacterial phyla and genera across stages of gastric carcinogenesis. **(A)** Box plots for the top 5 abundant bacterial phyla across disease progression. All the illustrated top 5 phyla with mean relative abundance > 1%. **(B)** Box plots for the top 10 abundant bacterial genera across disease progression. All the illustrated top 10 genera with mean relative abundance > 1%. p-values were determined by Kruskal–Wallis test.
