# Supplementary material for: Meta-analysis of mucosal microbiota reveals universal microbial signatures and dysbiosis in gastric carcinogenesis
Source: Oncogene. 2022 Jun 9;41(28):3599–610. doi: 10.1038/s41388-022-02377-9 (PMC9270228; doi:10.1038/s41388-022-02377-9)
Supplement: Supplementary file 2 — Figure S2 [file 41388_2022_2377_MOESM2_ESM.pdf]

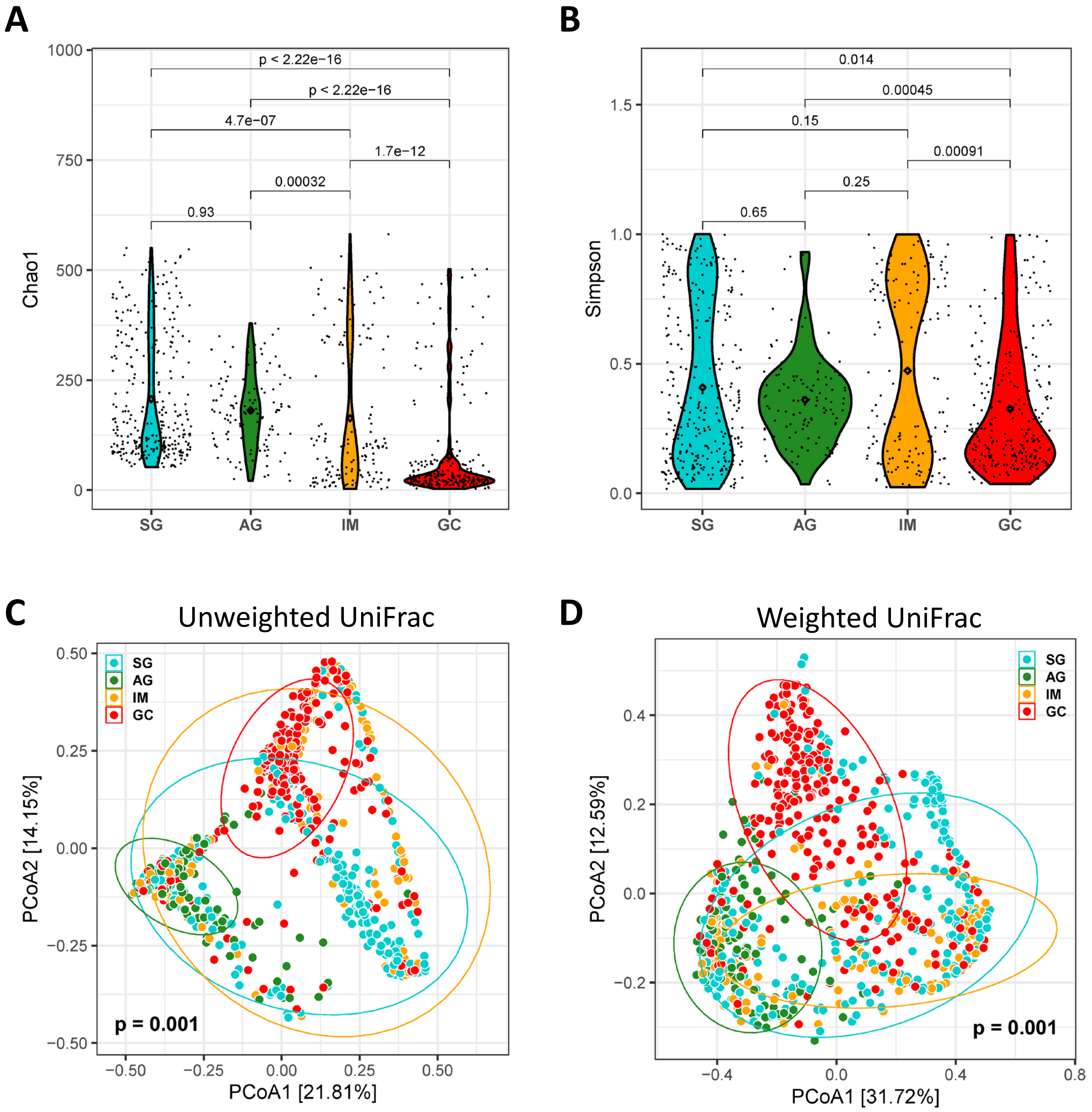

Figure S2. Bacterial diversities for the four group. **(A)** Alpha diversity estimated by Chao1 index for patients in each group. **(B)** Alpha diversity estimated by Simpson index for patients in each group. **(C)** PCoA with unweighted UniFrac distance for patients in each group. **(D)** PCoA with weighted UniFrac distance for patients in each group. p-values shown in PCoA plots were estimated by PERMANOVA.
