# Supplementary material for: Meta-analysis of mucosal microbiota reveals universal microbial signatures and dysbiosis in gastric carcinogenesis
Source: Oncogene. 2022 Jun 9;41(28):3599–610. doi: 10.1038/s41388-022-02377-9 (PMC9270228; doi:10.1038/s41388-022-02377-9)
Supplement: Supplementary file 3 — Figure S3 [file 41388_2022_2377_MOESM3_ESM.pdf]

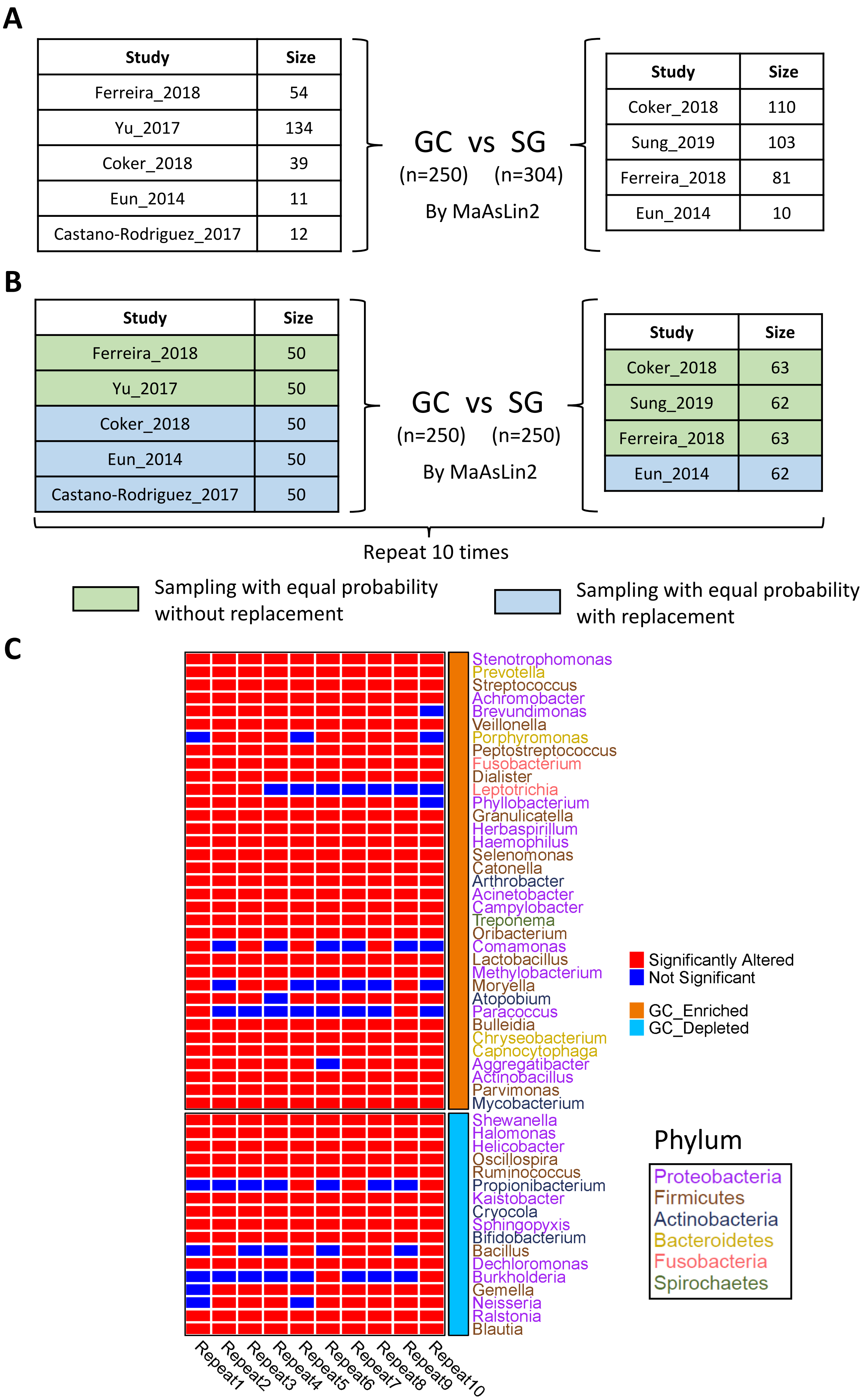

Figure S3. Verification of the differentially abundant bacteria between GC and SG by matching sample size for each involved study. **(A)** The general approach for identifying differentially abundant bacteria between GC and SG. This approach is corresponding to Figure 2A. **(B)** The approach for identifying differentially abundant bacteria between GC and SG by matching sample size for each involved study, with 10 repeats. **(C)** The heatmap for the significance of targeted bacteria identified in each repeat by matching sample size. Here the targeted bacteria were the 52 differentially abundant bacteria between GC and SG identified by the general approach, corresponding to Figure 2A.
