# Supplementary material for: Meta-analysis of mucosal microbiota reveals universal microbial signatures and dysbiosis in gastric carcinogenesis
Source: Oncogene. 2022 Jun 9;41(28):3599–610. doi: 10.1038/s41388-022-02377-9 (PMC9270228; doi:10.1038/s41388-022-02377-9)
Supplement: Supplementary file 4 — Figure S4 [file 41388_2022_2377_MOESM4_ESM.pdf]

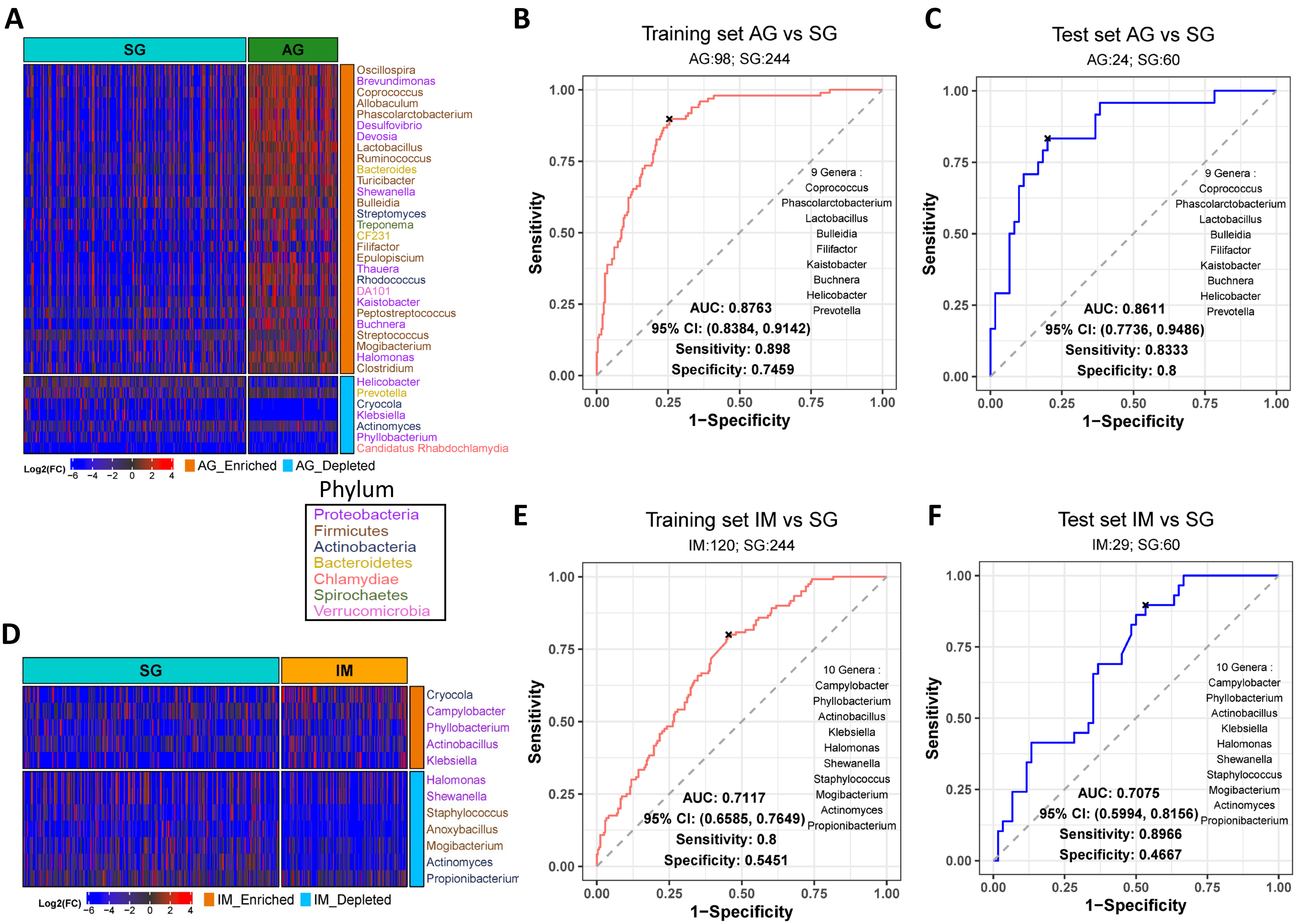

Figure S4. Differentially abundant bacteria for AG vs SG, IM vs SG and the related diagnostic genera markers. **(A)** Heatmap for the significant differentially abundant genera between AG and SG. **(B)** Receiver operating characteristic (ROC) analysis for the 9 genera markers with logistic regression model discriminating AG from SG in training set. **(C)** Receiver operating characteristic analysis for the same logistic regression model discriminating AG from SG in test set. **(D)** Heatmap for the significant differentially abundant genera between IM and SG. **(E)** Receiver operating characteristic analysis for the 12 genera markers with logistic regression model discriminating IM from SG in training set. **(F)** Receiver operating characteristic analysis for the same logistic regression model discriminating IM from SG in test set. The diagnostic genera markers were determined by backward stepwise selection algorithm from the significantly altered genera. The ratio of sample size of training set to that of test set was 8:2.
