# Supplementary material for: Meta-analysis of mucosal microbiota reveals universal microbial signatures and dysbiosis in gastric carcinogenesis
Source: Oncogene. 2022 Jun 9;41(28):3599–610. doi: 10.1038/s41388-022-02377-9 (PMC9270228; doi:10.1038/s41388-022-02377-9)
Supplement: Supplementary file 6 — Figure S6 [file 41388_2022_2377_MOESM6_ESM.pdf]

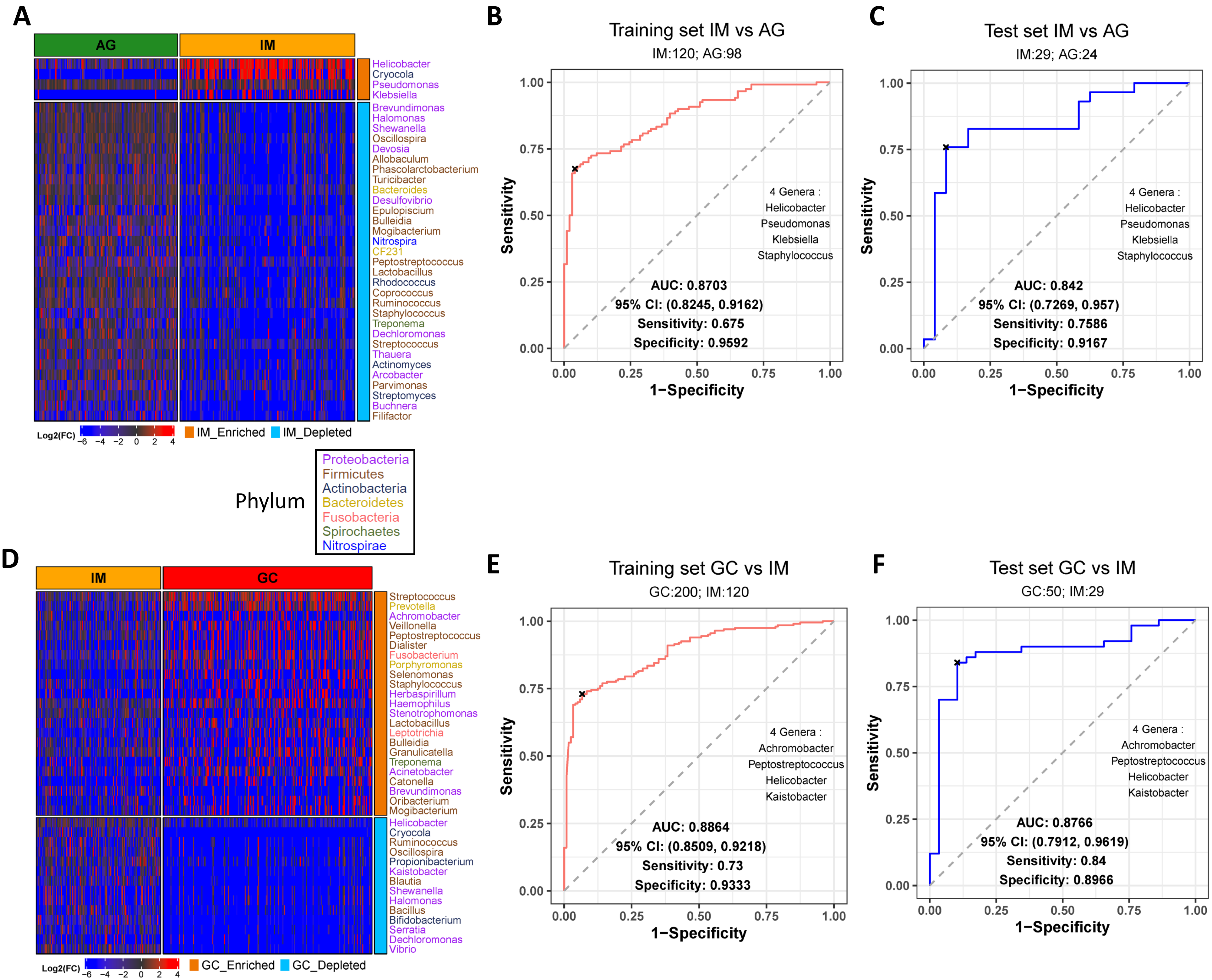


**Figure S6.** Differentially abundant bacteria for IM vs AG, GC vs IM and the related diagnostic genera markers. **(A)** Heatmap for the significant differentially abundant genera between IM and AG. **(B)** Receiver operating characteristic (ROC) analysis for the 4 genera markers with logistic regression model discriminating IM from AG in training set. **(C)** Receiver operating characteristic analysis for the same logistic regression model discriminating IM from AG in test set. **(D)** Heatmap for the significant differentially abundant genera between GC and IM. **(E)** Receiver operating characteristic analysis for the 4 genera markers with logistic regression model discriminating GC from IM in training set. **(F)** Receiver operating characteristic analysis for the same logistic regression model discriminating GC from IM in test set. The diagnostic genera markers were determined by backward stepwise selection algorithm from the significantly altered genera. The ratio of sample size of training set to that of test set was 8:2.
