# Supplementary material for: Meta-analysis of mucosal microbiota reveals universal microbial signatures and dysbiosis in gastric carcinogenesis
Source: Oncogene. 2022 Jun 9;41(28):3599–610. doi: 10.1038/s41388-022-02377-9 (PMC9270228; doi:10.1038/s41388-022-02377-9)
Supplement: Supplementary file 7 — Figure S7 [file 41388_2022_2377_MOESM7_ESM.pdf]

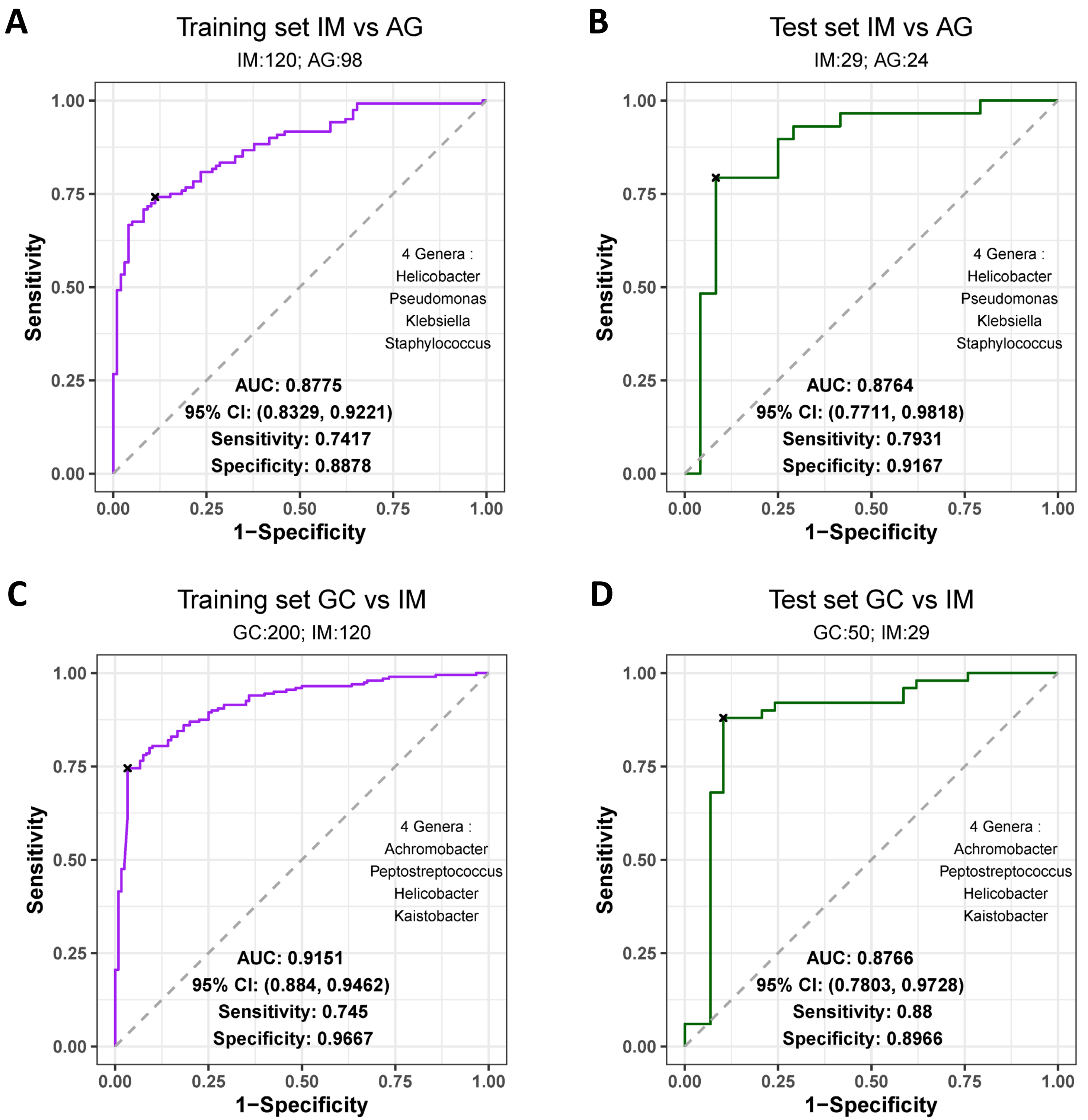

Figure S7. Validation of diagnostic genera markers for each discrimination by support vector machine (SVM) model. **(A)** Receiver operating characteristic (ROC) analysis for the 4 genera markers with SVM model discriminating IM from AG in the same training set. **(B)** Receiver operating characteristic analysis for the same SVM model discriminating IM from AG in the same test set. **(C)** Receiver operating characteristic analysis for the 4 genera markers with SVM model discriminating GC from IM in the same training set. **(D)** Receiver operating characteristic analysis for the same SVM model discriminating GC from IM in the same test set.
