# Supplementary material for: Meta-analysis of mucosal microbiota reveals universal microbial signatures and dysbiosis in gastric carcinogenesis
Source: Oncogene. 2022 Jun 9;41(28):3599–610. doi: 10.1038/s41388-022-02377-9 (PMC9270228; doi:10.1038/s41388-022-02377-9)
Supplement: Supplementary file 8 — Figure S8 [file 41388_2022_2377_MOESM8_ESM.pdf]

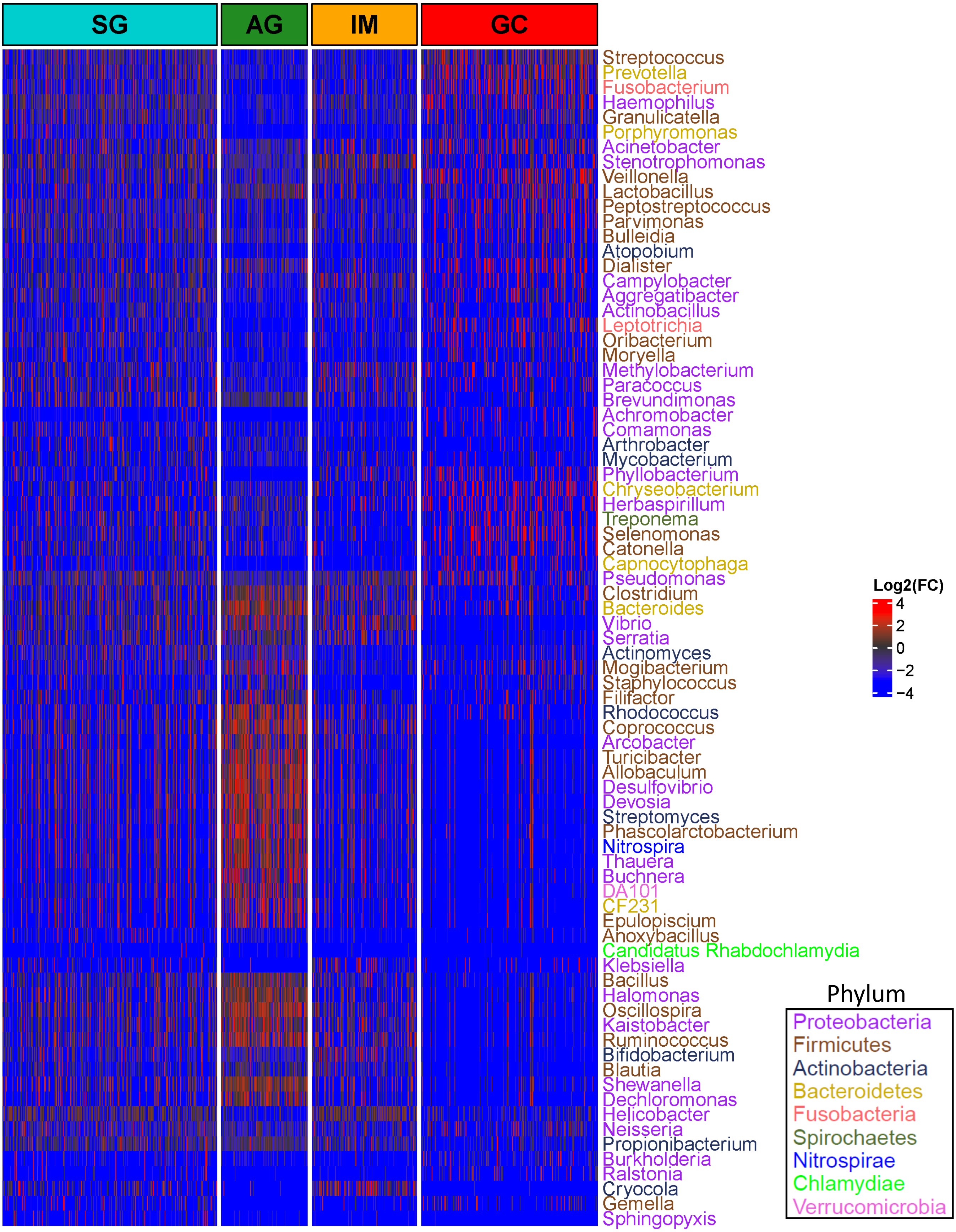

Figure S8. Heatmap for the significant differentially abundant genera for the four groups. The 79 genera shown here were the combination of significant differentially abundant genera for GC vs SG, AG vs SG, IM vs SG, IM vs AG and GC vs IM using MaAsLin2.
