# Supplementary material for: Meta-analysis of mucosal microbiota reveals universal microbial signatures and dysbiosis in gastric carcinogenesis
Source: Oncogene. 2022 Jun 9;41(28):3599–610. doi: 10.1038/s41388-022-02377-9 (PMC9270228; doi:10.1038/s41388-022-02377-9)
Supplement: Supplementary file 9 — Figure S9 [file 41388_2022_2377_MOESM9_ESM.pdf]

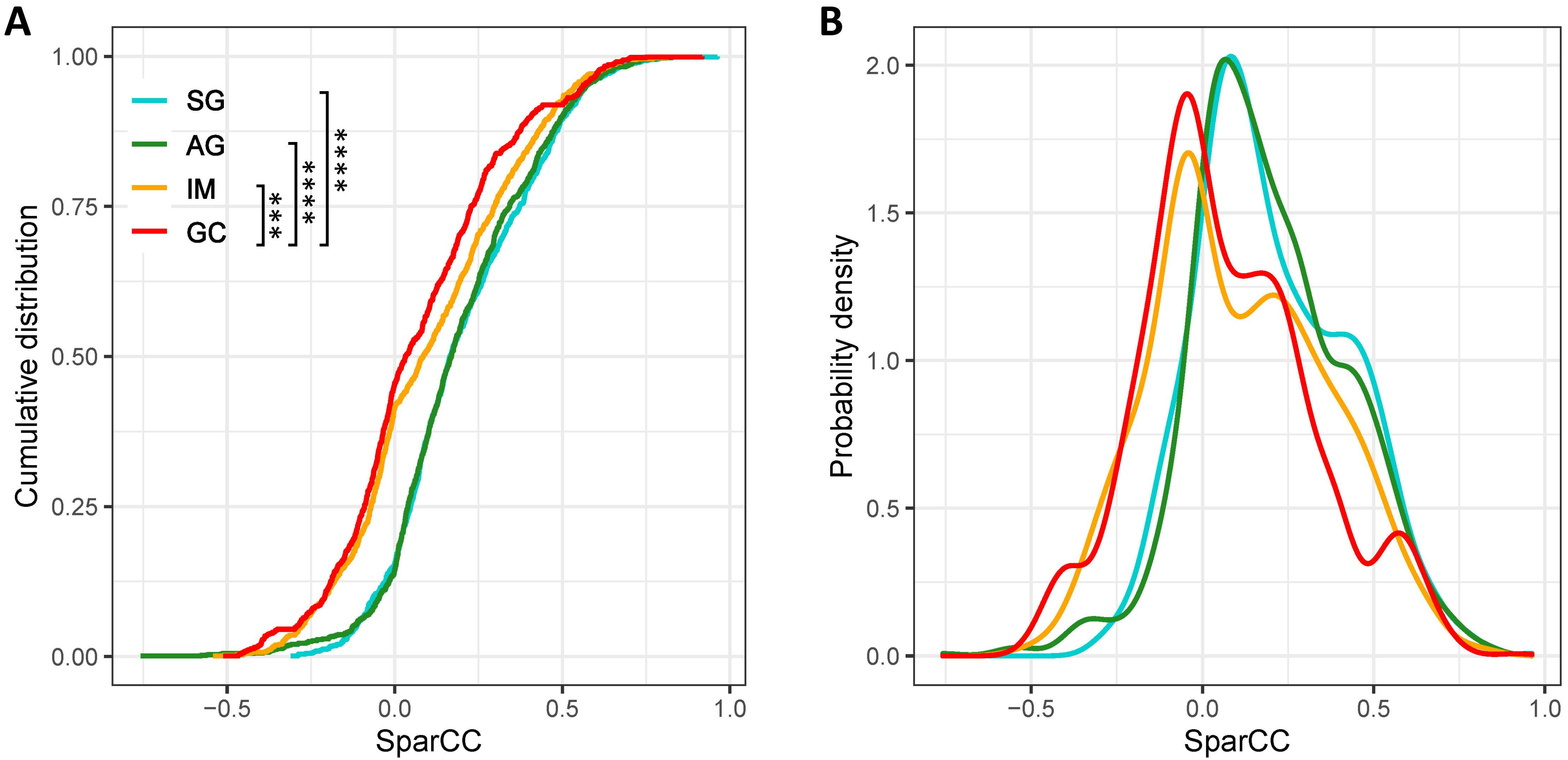

Figure S9. Correlation distributions of gastric cancer associated bacteria within each disease stage. **(A)** Cumulative distribution functions of SparCC correlations for gastric cancer associated bacteria within each disease stage. **(B)** Probability density functions of SparCC correlations for gastric cancer associated bacteria within each disease stage. p-values were determined by Kolmogorov-Smirnov test (***: $p<0.001$, ****: $p<0.0001$).
