# Supplementary material for: Meta-analysis of mucosal microbiota reveals universal microbial signatures and dysbiosis in gastric carcinogenesis
Source: Oncogene. 2022 Jun 9;41(28):3599–610. doi: 10.1038/s41388-022-02377-9 (PMC9270228; doi:10.1038/s41388-022-02377-9)
Supplement: Supplementary file 11 — Figure S11 [file 41388_2022_2377_MOESM11_ESM.pdf]

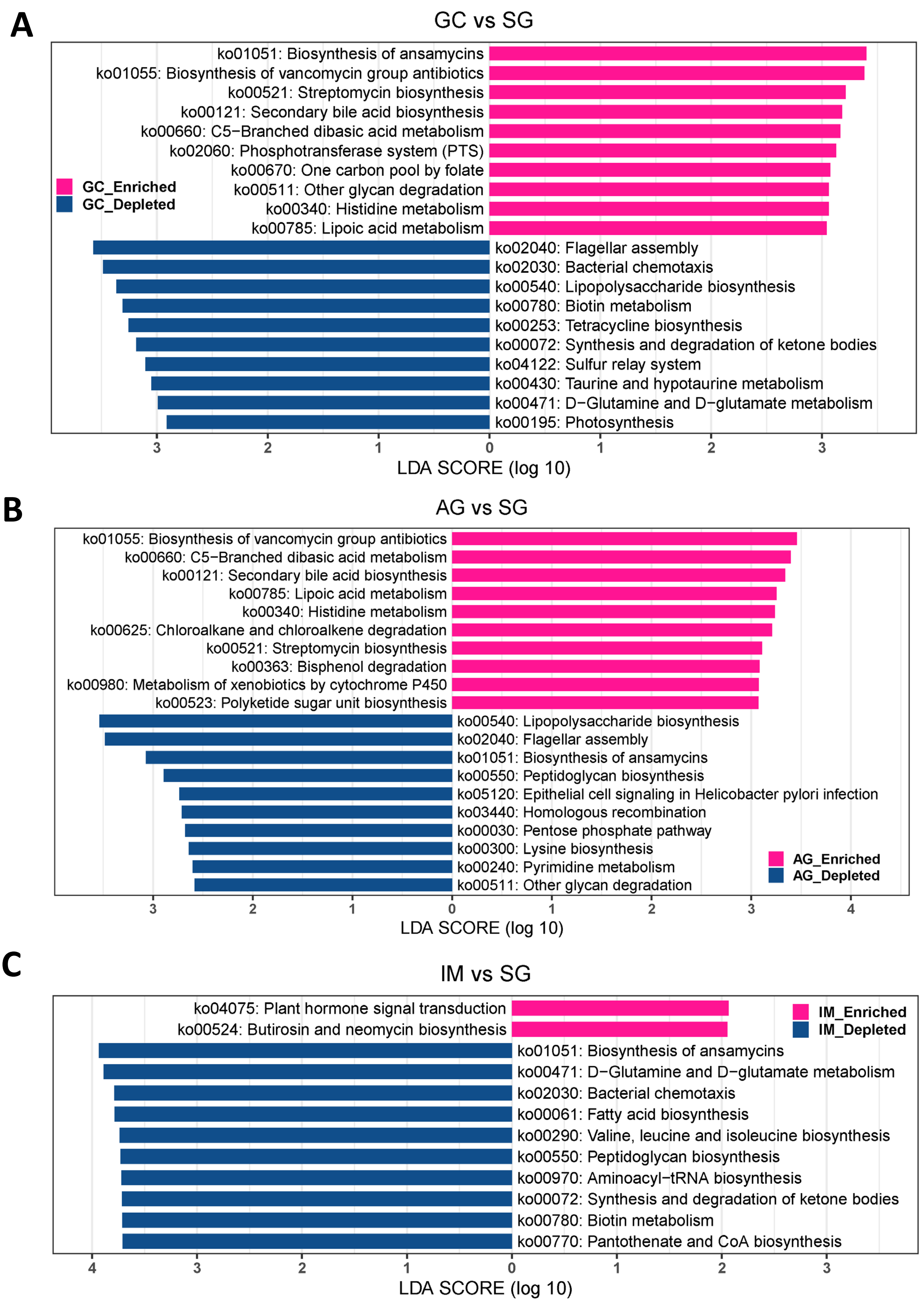

Figure S11. Predicted microbiota functional changes between stages of gastric cancer in KEGG pathways. **(A)** Bar plot of the top 10 significantly altered KEGG pathways between GC and SG. **(B)** Bar plot of the top 10 significantly altered KEGG pathways between AG and SG. **(C)** Bar plot of the top 10 significantly altered KEGG pathways between IM and SG. Significance was determined by Linear discriminant analysis (LDA) effect size (LEfSe) method with cutoff LDA score > 2 and p-value<0.05.
