# Supplementary material for: Meta-analysis of mucosal microbiota reveals universal microbial signatures and dysbiosis in gastric carcinogenesis
Source: Oncogene. 2022 Jun 9;41(28):3599–610. doi: 10.1038/s41388-022-02377-9 (PMC9270228; doi:10.1038/s41388-022-02377-9)
Supplement: Supplementary file 12 — Figure S12 [file 41388_2022_2377_MOESM12_ESM.pdf]

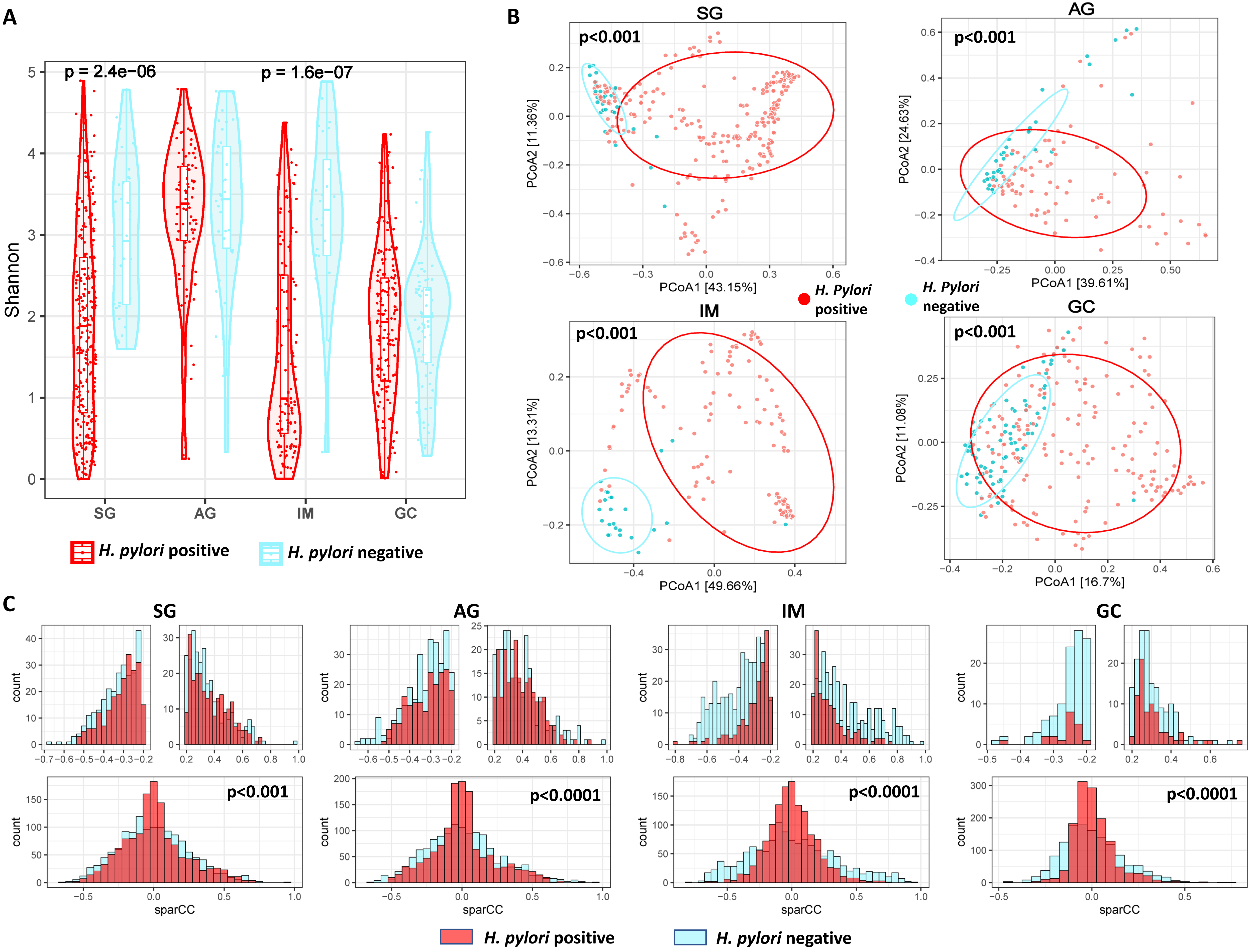

Figure S12. The influence of *Helicobacter pylori* in the microbiota community for each disease stage of gastric cancer progression. **(A)** Bacterial diversity (Alpha diversity) estimated by Shannon index for patients with different *H. pylori* status in each disease stage. p-values were obtained by Wilcoxon rank-sum test. **(B)** Principal coordinate analysis (PCoA, Beta diversity) for subjects in different *H. pylori* status for each disease stage. p-values were estimated by permutational multivariate analysis of variance (PERMANOVA). **(C)** Histograms of the distributions of SparCC correlation strengths for abundant bacteria with different *H. pylori* status in each disease stage. Genera with median of relative abundance > 0.1% were considered as abundant bacteria. p-values were obtained by Kolmogorov-Smirnov test.
