# Supplementary material for: Meta-analysis of mucosal microbiota reveals universal microbial signatures and dysbiosis in gastric carcinogenesis
Source: Oncogene. 2022 Jun 9;41(28):3599–610. doi: 10.1038/s41388-022-02377-9 (PMC9270228; doi:10.1038/s41388-022-02377-9)
Supplement: Supplementary file 10 — Figure S10 [file 41388_2022_2377_MOESM10_ESM.pdf]

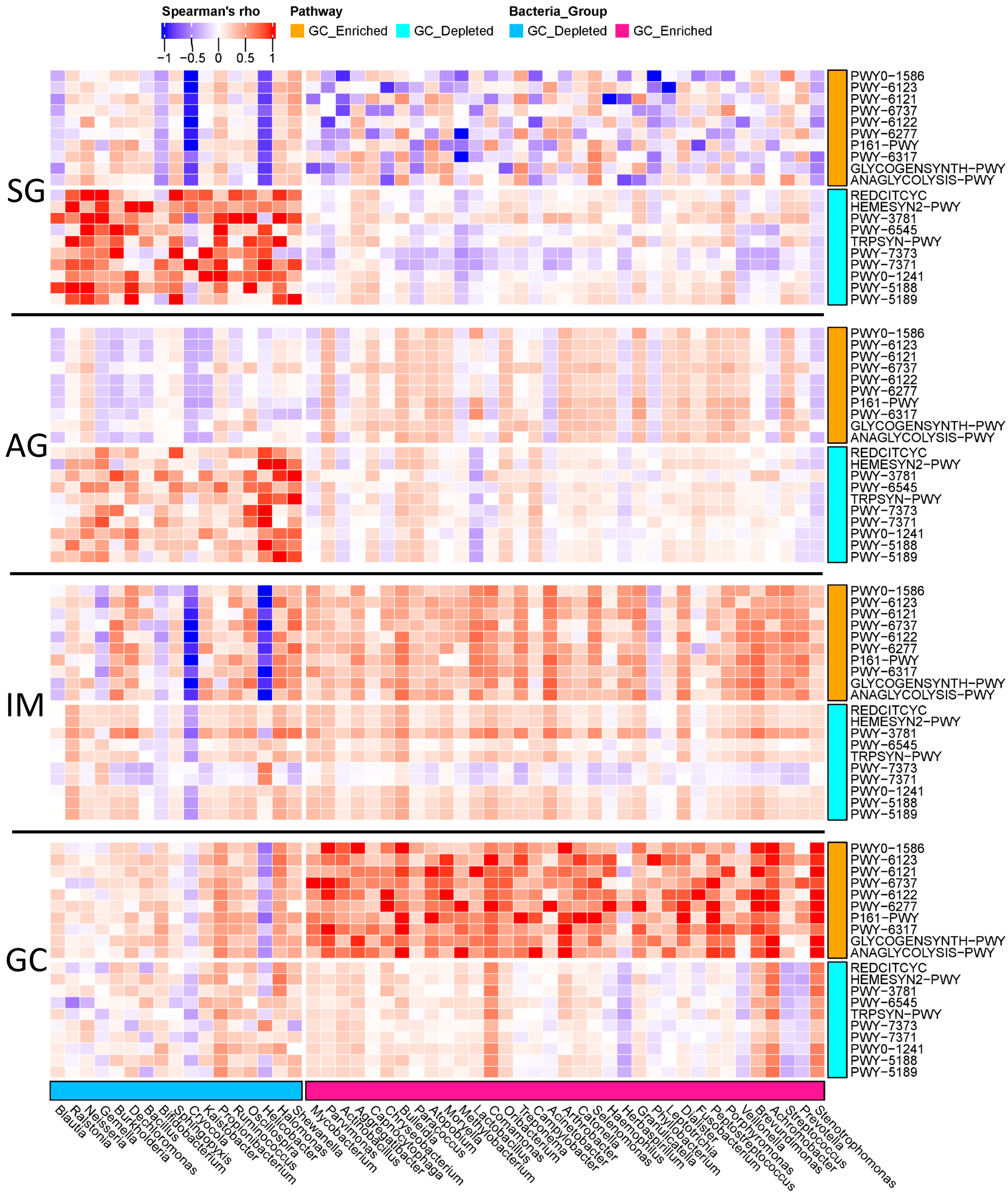

Figure S10. Heatmaps for the correlations of GC vs SG top altered functional MetaCyc pathways with GC vs SG significantly altered genera in each disease stage. Spearman's rank correlation coefficient (Spearman's rho) was used to measure the correlations of abundances of altered pathways with relative abundances of altered bacterial genera.
